# The Unmet Diagnostic and Treatment Needs in Large Cell Neuroendocrine Carcinoma of the Lung

**DOI:** 10.3390/curroncol30080523

**Published:** 2023-07-27

**Authors:** Catalin Buium, Serban Negru, Diana N. Ionescu, Mircea Dediu

**Affiliations:** 1 Department of Medical Oncology, Asociatia Oncohelp, 300239 Timisoara, Romania; serban.negru@umft.ro; 2 Department of Oncology, University of Medicine and Pharmacy “Victor Babes”, 300041 Timisoara, Romania; 3 Department of Pathology, BC Cancer, The University of British Columbia, Vancouver, BC V6B5M5, Canada; dionescu@bccancer.bc.ca; 4 Department of Medical Oncology, Sanador Clinical Hospital, 010991 Bucharest, Romania; mircea.dediu@sanador.ro

**Keywords:** large cell, neuroendocrine, lung cancer, pathology, molecular subtypes, diagnosis, treatment

## Abstract

Large cell neuroendocrine carcinoma of the lung (LCNEC) is currently classified as a rare lung cancer subtype, but given the high incidence of lung cancer, the overall number of cases is considerable. The pathologic diagnosis of LCNEC is mainly based on the microscopic appearance of the tumor cells, the mitotic rate, the amount of intra-tumoral necrosis, and the presence of positive neuroendocrine markers identified by immunohistochemistry. Recently, a subdivision into two main categories was proposed based on mutation signatures involving the RB1, TP53, KRAS, and STK11/LKB1 genes, into SCLC-like (small cell lung cancer-like) and NSCLC-like (non-small cell lung cancer-like) LCNEC. In terms of treatment, surgery is still the best option for resectable, stage I–IIIA cases. Chemotherapy and radiotherapy have conflicting evidence. Etoposide/platinum remains the standard chemotherapy regimen. However, based on the newly proposed LCNEC subtypes, some retrospective series report better outcomes using a pathology-driven chemotherapy approach. Encouraging outcomes have also been reported for immunotherapy and targeted therapy, but the real impact of these strategies is still being determined in the absence of adequate prospective clinical trials. The current paper scrutinized the epidemiology, reviewed the reliability of pathologic diagnosis, discussed the need for molecular subtyping, and reviewed the heterogeneity of treatment algorithms in LCNEC.

## 1. Introduction

LCNEC usually presents clinically and radiologically in a similar way to non-small cell lung cancer (NSCLC), as a peripheral lesion in the lung, with infrequent symptoms including cough, hemoptysis, pneumonia, though it is sometimes asymptomatic. Paraneoplastic syndromes are less common. LCNEC does not produce peptides as frequently as small cell lung carcinoma (SCLC). Sixty to eighty percent of cases initially present with lymph node metastases and approximately 40% present with distant metastases, the most frequent site being the liver. About 50% of the patients diagnosed with LCNEC present with operable disease. The prognosis is poor, as the 5-year survival is about 35% for all stages, almost overlapping the survival curves of SCLC. Even for early stage disease, the recurrence rate is high, and the overall survival is shorter than in the NSCLC subtypes [1,2,3].

## 2. Is LCNEC a Rare Type of Cancer?

LCNEC is a rare type of lung cancer, but given the high incidence of lung cancer in general, the overall number of cases of LCNEC is considerable.

LCNEC accounts for approximately 3% of resected lung carcinomas and is found primarily in older males aged > 65 years (male to female ratio of 17 to 1), with more than 90% of them being heavy smokers. Therefore, the leading documented risk factor for LCNEC is smoking, and its mutation signature is strongly associated with it, characterized by guanine to thymine and cytosine to adenine transversions. The high exonic mutation signature of LCNEC (8.6 non-synonymous mutations per one million base pairs) and high tumor mutation burden (TMB) further supports its strong relationship with smoking. Recent epidemiological studies showed an increase in the incidence of LCNEC, which is probably related to better pathologic diagnosis. There are approximately 1000–1500 new cases annually in the UK (out of the 46,000 new lung cancer cases) and approximately 4500–7000 in the USA (out of the 229,000 new lung cancer cases in 2018). The American Cancer Society estimated that there were about 236,740 new lung cancer cases in 2022, which allowed the recruitment of LCNEC patients into specific clinical trials [2,3,4].

## 3. Pathologic Diagnosis of LCNEC and Its Limitations

In the latest World Health Organization (WHO) Classification of Thoracic Tumors (2021), there are four major types of neuroendocrine tumors of the lung: carcinoid, atypical carcinoid, SCLC, and LCNEC. Tumorlets and diffuse idiopathic pulmonary neuroendocrine cell hyperplasia (DIPNECH) are listed as pre-neoplastic lesions. Carcinoid, atypical carcinoid, small cell carcinoma (SCLC), and large cell neuroendocrine carcinoma (LCNEC) do not represent a continuum, and the carcinoids (typical or atypical), while biologically related, are epidemiologically and molecularly distinct from the more aggressive SCLC and LCNEC [5].

The WHO’s proposed defining criteria for LCNEC are the same today as their introduction in 1991, classifying LCNEC of the lung as a neuroendocrine carcinoma with a SCLC clinical phenotype (aggressive behavior, high recurrence rate following definitive local treatment, and similar metastatic pattern). The histology is challenging and usually requires the examination of a resection specimen for a definitive diagnosis, as the WHO recommends. Therefore, the diagnosis is seldom made in advanced or metastatic disease. The increasing need for larger samples for molecular studies involving lung cancer patients allowed for more LCNECs to be diagnosed in smaller samples and at advanced stages. LCNEC cells are typically more than three times the diameter of small lymphocytes, but cell size is just one of the morphologic criteria used in differentiating LCNEC from SCLC, despite their names [1,2,5].

On conventionally stained slides (hematoxylin and eosin), some specific morphologic characteristics are required to diagnose a neuroendocrine neoplasm. The characteristic neuroendocrine architecture with various organoid growth patterns (rosette, palisading, ribbons, festoons, trabeculae) is usually observed at a low power magnification. At a higher power, the nuclear details are also highly suggestive of neuroendocrine differentiation, with finely granular, speckled chromatin (“salt and pepper”), and often with vesicular nuclear chromatin and nucleoli. Cellular pleomorphic and cytological atypia vary among the lung’s four NET, but are not distinguishable enough. The significant diagnosis determinants which, by the WHO definition, separate these entities are the presence and amount of necrosis and the number of mitoses. For instance, in LCNEC, extensive necrosis is often present in confluent areas, and a median of >70 mitoses/2 mm^2^ is typical, although the WHO definition requires a mitotic count of only >10 mitosis/2 mm^2^. Neuroendocrine markers by immunohistochemistry (synaptophysin, chromogranin A, CD56) are required for the diagnosis of LCNEC. There are several new immunohistochemical stains for neuroendocrine differentiation, such as Insulinoma-associated protein 1 (INSM1) and Achaete-scute homolog-1 (ASCL-1), which have been described as markers with nuclear expression. The sensitivity and specificity of INSM1 for diagnosing lung NETs are excellent and, thus, it is often used in clinical practice [6,7].

The primary differential diagnosis for LCNEC is SCLC and atypical carcinoid. The similar neuroendocrine features and biomarker expression makes the differentiation between SCLC and LCNEC challenging, the latter being distinguished mainly by subtle morphologic features. No genetic or immunohistochemical marker can reliably differentiate between LCNEC and SCLC. Abundant cytoplasm and/or the presence of nucleoli favor the LCNEC diagnosis. In addition, a cell size larger than 25 microns (µm) strongly suggests LCNEC. Loss of expression of the Retinoblastoma gene-1 (RB1) is helpful in equivocal cases, as RB1 is lost in more than 95% of SCLCs and only in approximately 50% of LCNECs, but this immunostain is rarely used clinically. Areas of extensive necrosis, prominent mitotic activity, and a higher Ki67 are the main criteria to distinguish LCNEC from atypical carcinoids. However, the diagnosis is often impossible in small samples with borderline Ki67 of >25%, as seen in the newer entity of “supracarcinoids” [1,3,8]. Such tumors, with a mildly elevated mitotic count, are relatively common in metastatic settings and are only rarely encountered in samples from primary lung tumors. Paraneoplastic syndromes are frequently associated with SCLC and uncommonly seen in carcinoids, but they are rare in AC and LCNEC. LCNECs are mixed tumors in up to 20% of cases, and show a small-cell (SCLC) or non-small-cell lung cancer (NSCLC) component.

**Figure 1:**
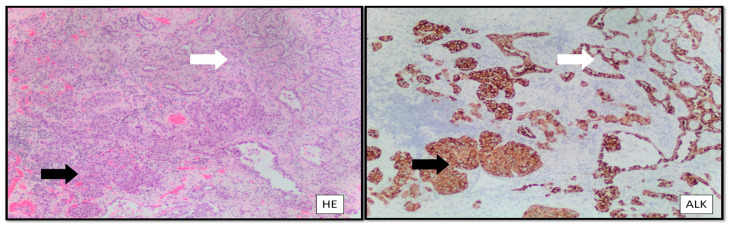
Combined large cell neuroendocrine carcinoma and adenocarcinoma of the lung with ALK rearrangement. White arrow: Adenocarcinoma. Black arrow: LCNEC. HE: Hematoxylin and Eosin; ALK: anaplastic lymphoma kinase.

LCNEC needs to be distinguished from solid-type adenocarcinoma (Figure 1), which may express an isolated neuroendocrine marker (no more than one) in 10–20% of cases, as compared with at least two IHC neuroendocrine markers in the majority of LCNEC cases. No prognostic or therapeutic relevance is associated with this single neuroendocrine marker identification in adenocarcinoma. The newer entity of thoracic SMARC-A4-deficient undifferentiated tumor can express synaptophysin and mimic LCNEC clinically and pathologically. Still, the proof of SMARC-A4 (BRG1) loss by immunohistochemistry is reliable and sufficient for its diagnosis. Less specific, but helpful in the differential diagnosis, is the lack of claudin 4 expression and low to absent keratin staining in SMARCA4-deficient thoracic tumors [6,7].

We should add for completeness that other entities, primary or metastatic to the lung, enter the differential diagnosis of LCNEC, including basaloid squamous cell carcinoma, Merkel cell carcinoma, and some sarcomas and lymphomas, but the details regarding separating them from LCNEC, given their rarity, is beyond the scope of this paper. 

## 4. Molecular Subtypes of LCNEC

In 2016–2018, studies on LCNEC described two major groups: SCLC-like and NSCLC-like. The division was based on specific mutation signatures involving the RB1, TP53, KRAS, and STK11/LKB1 genes [1].

Rekhtman and colleagues identified two major molecular subsets of LCNEC by performing next-generation sequencing (NGS) on 45 histologically pure LCNECs. The first group was the SCLC-like subset, which accounted for 40% of the cases and was characterized by the alteration of protein p53 (TP53) and RB1. The second group, the NSCLC-like subset (56) characterized by STK11, KRAS, and KEAP1 mutations, lacked the alterations of RB1 and TP53. Both subsets presented a higher tumor mutation burden than NSCLC and SCLC, suggesting the possible sensitivity of LCNEC to immunotherapy [8].

George and colleagues analyzed 75 cases of LCNEC. Two distinct subgroups were identified: type I (37%), with TP53 and STK11/KEAP1 alterations, being similar to NSCLC, and type II (42%), similar with SCLC, which presents with the inactivation of TP53 and RB1 and also an upregulation of the immune-related pathways [9].

Miyoshi and colleagues analyzed the genomic profiles of both LCNEC and SCLC, and they showed that the profile was not so different, with a lower alteration of RB1 in LCNEC and alterations in the PI3K/AKT/mTOR pathway (15%), KRAS (6%), FGFR1 (5%), and EGFR (1%), suggesting potential targeted therapies for this challenging type of lung cancer. The study also found a median genetic alteration concordance rate of 71% among the 10 cases of combined LCNEC and NSCLC [10].

These studies identified the subtypes of LCNEC with their genomic profiles and potential targets. They found correlations between the subtypes and the response to chemotherapy, with impacts on prognosis and treatment, suggesting that genomic characterization should be performed, whenever possible, in advanced cases of LCNEC [1].

## 5. Role of Surgery

LCNEC is diagnosed in resectable stages (I–IIIA) in roughly 25% of cases [3].

Surgery is independently associated with a better overall survival (OS), and lobectomy/bilobectomy has better outcomes than pneumonectomy or segmentectomy [11]. A poorer prognosis after surgery is associated with advanced stage and nodal involvement, and with the expression of chromogranin A and/or CD56 [12].

Tumor recurrence after surgery is 64% within one year and 91% within three years. Thus, adjuvant chemotherapy aims to extend the progression-free survival (PFS). There is only one prospective study that evaluated the efficacy of the combination of cisplatin and etoposide after patients who received lobectomy with lymph node dissection, but the control group was derived from retrospective data. The five-year OS was 88.9% vs. 47.5% in favor of the adjuvant arm [13]. A multivariate analysis also showed that platinum-based adjuvant chemotherapy may improve the prognosis significantly [14].

Wakeam and colleagues found, in a retrospective study, that adjuvant chemotherapy received within three to six months after surgery was associated with better OS. The difference was most significant in patients with tumors larger than 3 cm (5-year OS 59.8% vs. 42.1%). For the patients presenting with tumors smaller than 2 cm and the patients for whom the treatment started six months of more after the surgery, there was no benefit [15].

Kunwei et al. studied patients with early stage LCNEC (T1N0M0) from 2004 to 2016; most of them (59.5%) received lobectomy and they had a better survival than those cases in which sublobar resection was the choice (37% vs. 51.9% at 3 years). Better survival outcomes were seen in patients with at least four resected lymph nodes. On the other hand, the survival in patients with tumors smaller than 2 cm was not improved by giving adjuvant chemotherapy (*p* = 0.658). Age and lobectomy were independent prognostic factors (*p* = 0.000), as multivariate analysis showed [16].

Sixty-three patients diagnosed with LCNEC were evaluated in a retrospective study. The study found that perioperative chemotherapy was associated with better OS than surgery alone; a better five-year OS was observed in non-triple-positive patients (with tumors that are not immunoreactive to synaptophysin, chromogranin A, CD56), but no difference was found in the triple-positive group. Chromogranin A positive tumors had no benefit from adjuvant treatment [17].

The evaluation of the mammalian Notch family ligands delta-like 3 expression (DLL3) may be useful for the differentiation the patients who will benefit from adjuvant chemotherapy. DLL3 was evaluated in 70 patients with LCNEC undergoing resection, of whom 23 patients (32.9%) received platinum-based adjuvant chemotherapy. In DLL3 expression-positive tumors, there was no significant difference in the five-year overall survival (OS) or recurrence-free survival (RFS) between patients with and without adjuvant chemotherapy (OS: 58.3% vs. 35.7% *p* = 0.36, five-year RFS: 41.7% vs. 35.7% *p* = 0.74). In contrast, significantly greater five-year OS and RFS rates were observed among the DLL3-negative tumors, comparing patients with and without adjuvant chemotherapy (five-year OS: 90.0% vs. 26.9% *p* < 0.01, five-year RFS: 80.0% vs. 21.7% *p* < 0.01) [18].

One can conclude that, for resectable, stage I–IIIA LCNEC, surgery remains the cornerstone of treatment, along with cisplatin-based chemotherapy as a reasonable adjuvant recommendation.

## 6. Role of Chemotherapy in Advanced Stages

There was only one prospective study that evaluated the efficacy of cisplatin plus etoposide. It included 42 patients with metastatic disease and showed a median overall survival (mOS) of 7.7 months, which is slightly worse than the mOS reported for patients with SCLC treated with the same combination of chemotherapy [19].

The standard SCLC regimens seem to have better outcomes compared with the chemotherapy used in NSCLC, in an unselected patient population. For example, in a retrospective study, the overall response rate (ORR) was 73% vs. 50%, the median progression-free survival (mPFS) was 6.1 vs. 4.9, and the mOS was 16.5 vs. 9.2 months for the SCLC-like regimens compared with the NSCLC-like ones. However, the differences were statistically not significant, given the small number of patients [20]. A retrospective study of 294 LCNEC patients found the opposite results. First-line platinum-based combined chemotherapy was clustered as “NSCLC-t”, using gemcitabine, docetaxel, paclitaxel, or vinorelbine; “NSCLC-pt”, using pemetrexed treatment only; and “SCLC-t”, which consisted of etoposide chemotherapy. The mOS for NSCLC-t chemotherapy was 8.5 months. This is significantly better than patients treated with NSCLC-pt, with a mOS of 5.9 months, hazard ratio (HR) of 2.51, and 95% CI 1.39–4.52; *p* = 0.002, and also than patients treated with SCLC-t chemotherapy had a mOS of 6.7 (5.0–8.5) months (HR 1.66, 95% CI 1.08–2.56; *p* = 0.020) [21].

Some attempts have been made to change the chemotherapy regimens in accordance with the newly described LCNEC subtypes. Sixty-three patients with LCNEC were classified into small-cell lung cancer (SCLC)-like and non-small-cell lung cancer (NSCLC)-like LCNEC in a retrospective study, based on genomic features derived from tumor DNA and/or cfDNA. In SCLC-like LCNEC patients, the platinum etoposide (PE) regimen showed a superior mPFS compared with the NSCLC chemotherapy, at 8.3 vs. 3.0 months; *p* = 0.0013), whereas for patients with NSCLC-like LCNEC, SCLC regimens also appeared to be better, or at least not inferior than NSCLC. However, when a more comprehensive genomic alteration profile was used for LCNEC subtyping (including RB1 mutation or loss, PTEN loss/mutation, FGFR1/FGFR4 mutation/amplification, and TP53 loss), pemetrexed-platinum was the better regimen, suggesting that the use of larger NGS panels should be considered to identify the subgroups of patients who may benefit from different regimens [22].

Modest activity was reported for amrubicine in a second-line setting. The overall response rate (ORR) was 27.7%, mPFS 3.1 months, and mOS 5.1 months [23].

The conflicting results and the results based on a retrospective assessment of small patient samples highlight the need for more prospective trials. Overall, chemotherapy alone offers small OS benefits. Whether or not the case is SCLC-like, cisplatin plus etoposide chemotherapy remaining the preferred regimen is a matter of debate. The use of LCNEC genomic subtype-driven chemotherapy needs more convincing prospective data to be implemented in current clinical practice.

## 7. Role of Radiotherapy

Radiotherapy also brings conflicting evidence; a large retrospective study (*n* = 3371) included patients with stage I disease and compared surgery (96%) with stereotactic body radiation therapy (SBRT) (4%). The OS at five years was 50% versus 27% (HR = 0.7). For stage II–IIIA, surgery has a better OS than definitive chemoradiation, although 40% of the patients received induction/adjuvant chemotherapy [24]. Patients that are unsuitable for surgery benefit more from chemoradiation than chemotherapy alone. Additionally, in the postoperative setting, radiotherapy had a detrimental effect (mOS 27 vs. 44 months with surgery alone) [1]. Michael et al. showed that radiotherapy could benefit stage I–II cases with positive margins, and stage III patients treated with both surgery and radiotherapy had the best survival outcome [25].

Approximately 20% of the cases of LCNEC are diagnosed as locally advanced (IIIB–C). Definitive chemo-radiotherapy was compared in a retrospective study (*n* = 5797) with chemotherapy alone, and the study found an mOS of 16.1 vs. 11.9 months [26]. The results were confirmed by Gu and colleagues [27]; Shimada et al. found a higher objective response rate for chemo-radiotherapy than chemotherapy alone (86% vs. 61%) [28].

There are limited data on the role of prophylactic cranial radiation (PCI) in early stage LCNEC. PCI is used significantly less in LCNEC as compared to SCLC, a retrospective study reported. A study of the LCNEC population using the SEER database noted a rate of brain metastases of 19.2% as compared to 16.7% in SCLC. LCNEC may be susceptible to brain irradiation, but further clinical trials are needed to clarify the role of PCI in this setting [29].

In the absence of prospective clinical trials, the recommendation for unresectable stage III disease is chemo-radiotherapy with four cycles of cisplatin plus etoposide [1].

## 8. Role of Immunotherapy

Immune checkpoint inhibitors (ICI) are approved in both SCLC and NSCLC, but their role in LCNEC management is yet to be defined.

Programmed death ligand-1 (PD-L1) is expressed in tumor cells (TC+) in around 10–25% of cases. Some studies reported a high positivity of PD-L1 staining in the intra-tumoral immune cells (IC+) rather than in the tumor cells (TC+). In a series of 68 patients, for instance, 11% and 75% of the tumor samples were TC+ and IC+, whereas 66% had a TC−/IC+ profile [30,31]. This profile is different when compared with the ~60% TC+ observed for NSCLCs [32] and 15% TC+ and 22% IC+ for SCLC, despite using the same 22C3 antibody [33].

The data are highly controversial regarding the prognostic significance of the PD-L1 expression in LCNEC. Some studies reported that PD-L1 expression (TC+ with or without IC+) is associated with a favorable prognosis [34].

On the contrary, other studies showed a poorer outcome in the PD-L1-positive tumor cells but with negative immune cells (TC+/IC−) as compared with the PD-L1-negative tumor cells and with positive immune cells (TC−/IC+) [30].

These controversial data point to the fact that prospective trials based on a standardized PD-L1 assessment are needed to clarify the prognostic value of PD-L1 expression in LCNEC.

Concerning ICI therapy, encouraging results were initially communicated in some anecdotal case reports. Larger retrospective studies support the individual case data. Using the National Cancer Database, a retrospective analysis was performed in 661 LCNEC cases, of whom 37 were treated with ICI. The use of ICI was associated with improved overall survival (log-rank *p* = 0.0018), and it remained statistically significant even after multivariate analysis (HR = 0.64, *p* = 0.0164). The patients in the ICI group had 12- and 18-month survival rates of 34.0% and 29.1%, as compared to 24.1% and 15.0% in the non-ICI group. Moreover, a propensity score matching analysis showed a non-significant trend (*p* = 0.0733) in favor of the ICI group in terms of overall survival [35]. A real-world retrospective study evaluated 125 LCNEC patients, of whom 41 received ICI (group A) and 84 did not (group B) showed similar trends in the favor of the ICI group. The median OS was 12.4 mo and 6.0 mo in groups A and B, respectively (log-rank test, *p* = 0.02). For ICI administration, the adjusted HR for OS was 0.58, *p* = 0.04. In a propensity score matching analysis, the median OS was 12.5 mo and 8.4 mo in matched groups A and B, respectively (log-rank test, *p* = 0.046) [36].

One can conclude that the role of immunotherapy in LCNEC is promising, but remains poorly defined. The retrospective series provide only hypothesis-generating data. Clarifications are expected from the results of the ongoing specifically designed clinical trials (Table 1).

## 9. Role of Targeted Therapy

Pure LCNEC rarely presents with targetable driver mutations, but they are more often detected in mixed forms of LCNEC-adenocarcinoma. In addition to TP53 and RB1 alterations, which are diagnostically relevant, the prevalence of potentially actionable mutations is less common in LCNEC compared with classical NSCLC. For instance, a series of 467 cases found EGFR exon 19 deletion, along with EGFR L858R point mutations, in only 1% of cases, whereas ALK fusions were identified in 1.7% of patients, all being classified as NSCLC-like subtype. KRAS G12C was identified in only 2.9%, whereas RET fusion, NTRK fusion, and BRAFV600E were not detected at all [37].

In a Chinese study, 26.3% (15/57) of patients diagnosed with LCNEC presented alterations in classical NSCLC driver genes, including missense mutations in KRAS, EGFR, and BRAF and amplifications in KRAS, EGFR, ERBB2, and MET, highlighting the potential of using targeted therapy. In some cases (7%), the analysis also identified an actionable EGFR p.L858R. Two of these patients received gefitinib. They achieved partial remission (PR), with one of them achieving a PFS of 15 months [38].

A few reported cases of EGFR mutations and ALK translocations were treated with selective TKIs, but the results were controversial. Treatment with crizotinib for LCNEC with EML4-ALK rearrangement proved to be inefficient according to two case reports [39,40], but one case with PLB1-ALK rearrangements responded crizotinib [41]. Another case report presented a partial response to alectinib in a combined adenocarcinoma/LCNEC tumor with metastases to the bone [42].

A case report from 2013 presented an 84-year-old patient diagnosed with metastatic LCNEC with EGFR exon 19 deletion. The patient received orally icotinib at a dose of 125 mg three times per day, up to a total of eight months of treatment. During these eight months, the patient reported a significant reduction in back pain and coughing. Also, the computed tomography scans of the chest revealed a partial remission after one month. This case highlighted the possibility of targeting EGFR mutations in LCNEC [43].

Despite the lack of supporting data, some other molecular alterations may offer rational approaches to LCNEC. As previously mentioned, DLL3 expression is positive in around 37% of LCNEC cases. Positive expression was associated with lack of sensitivity to chemotherapy in an adjuvant setting [18]. Despite negative outcomes with rovalpituzumab (a specific anti-DLL3 antibody drug conjugate) in later stage SCLC, this molecular abnormality may offer a potential target for further drug development in LCNEC [44]. Homologous recombination pathway mutations have also been identified in LCNEC and are associated with a longer trend in PFS following platinum-based chemotherapy [38]. Emerging evidence also suggests that tumors with mutations in the homologous recombination pathway process are sensitive to treatments that target the DNA repair pathway, such as the PARP inhibitors [45,46]. Therefore, evaluation of PARP inhibitors in the homologous recombination pathway-deficient LCNEC subset may be worthwhile.

In the neuroendocrine lung tumors, overactivation of the PI3K/AKT/mTOR pathway has been documented [47]. This offers the perspective of obtaining a clinical benefit by specifically blocking this aberrant pathway. In a phase II study, the combination of everolimus (an mTOR inhibitor) with paclitaxel and carboplatin was evaluated. The overall response rate (ORR) was 45%, with a median PFS of 4.4 months, and a median OS of 9.9 months. The premature closure of the trial precluded a clear interpretation of these data [48].

Somatostatin targeting agents, which include somatostatin analogues and peptide receptor radionuclide therapy (PRRT), are effective in well-differentiated neuroendocrine tumors. Their efficacy in LCNEC is yet to be determined. Case reports described a good response to PRRT in patients diagnosed with pulmonary LCNEC with positive somatostatin receptor scintigraphy [49].

TrkB (tropomyosin-related kinase B) and its ligand BNDF (brain-derived neurotrophic factor) play a key role in tumor progression in many other types of cancer. Odate et al. designed a study to investigate BDNF/TrkB signaling for neuroendocrine tumors of the lung: SCLC and LCNEC. The study found that TrkB and BDNF expression levels were significantly higher in LCNEC than in SCLC. Also, there was a correlation between the two expressions in LCNEC, but not in SCLC. There were only 11 patients diagnosed with LCNEC analyzed in the study. In order to define the correlation between BDNF/TrkB expression and prognosis in LCNEC, further investigations are required, but this may be a diagnostic aid for differentiation between the two entities (LCNEC and SCLC), and also a target for possible specific therapy [50].

One can conclude that, even though some targetable genomic alterations were identified in LCNEC, recent experience with targeted agents has been extremely limited.

## 10. Conclusions

LCNEC is a challenging disease, which requires further understanding of its biology for a more accurate subclassification and taxonomy. Although patients are treated with modified SCLC-specific regimens, with similar or sometimes worse outcomes, the recent findings regarding LCNEC molecular subtypes suggest that molecular features could guide the treatment approach. Limited data showed the efficacy of immunotherapy, which showed a radiological and clinical response in some patients pre-treated with platinum-based chemotherapy. Targeted therapies usually showed moderate efficacy, but there is a definite, unmet need for a large prospective clinical trial in this subtype of lung cancer.

## Figures and Tables

**Table 1:** Current clinical trials focusing on immunotherapy (according to https://clinicaltrials.gov, accessed on 24 October 2022).

NCT Number	Drug and Condition	Status
03901378	Pembrolizumab in GI-tract and LCNEC of the lung	Withdrawn
03976518	Atezolizumab in NSCLC: rare histologies	Recruiting
03728361	Nivolumab and temozolomide in refractory SCLC or advanced neuroendocrine cancer	Active, not recruiting
03305133	Evaluation of PD-L1 expression in LCNEC	Completed
03591731	Nivolumab ± ipilimumab in advanced/refractory GI-tract and LCNEC of the lung	Active, not recruiting
02834013	Nivolumab and ipilimumab in rare tumors (including LCNEC of the lung)	Recruiting
